# A randomized controlled trial on the effect of blue-blocking glasses compared to partial blue-blockers on melatonin profile among nulliparous women in third trimester of the pregnancy

**DOI:** 10.1016/j.nbscr.2021.100074

**Published:** 2021-12-29

**Authors:** Randi Liset, Janne Grønli, Roger Ekeberg Henriksen, Tone Elise Gjøtterud Henriksen, Roy Miodini Nilsen, Ståle Pallesen

**Affiliations:** a Department of Psychosocial Science, Faculty of Psychology, University of Bergen, Bergen, Norway; b Department of Biological and Medical Psychology, Faculty of Psychology, University of Bergen, Bergen, Norway; c Faculty of Health and Social Sciences, Western Norway University of Applied Sciences, Bergen, Norway; d Valen Hospital, Division of Mental Health Care, Fonna Local Health Authority, Valen, Norway; e Norwegian Competence Center for Sleep Disorders, Haukeland University Hospital, Bergen, Norway; f Optentia, The Vaal Triangle Campus of the North-West University, Vanderbijlpark, South Africa

**Keywords:** Blue light, Blue blocking glasses, Melatonin, Sleep, Pregnancy, RCT

## Abstract

**Objective:**

In pregnancy melatonin regulates circadian rhythms, induce sleep, and has a neuroprotective positive effect on fetal development. Artificial blue light in the evening delays and suppresses melatonin production. Thus, we investigated the effect of blocking blue light on the melatonin profile.

**Methods:**

A randomized controlled trial (n=30 blue-blocking glasses vs. n=30 control glasses with partial blue-blocking effect) including healthy nulliparous pregnant women in the beginning of the third trimester. Salivary melatonin and subjective sleep were measured before and after two weeks of intervention/control condition. Saliva was sampled at 30-min intervals from 3 h before normal bedtime. Melatonin onset was set at 4.0 pg/ml.

**Results:**

Due to missing data melatonin onset was estimated for 47 participants. At posttreatment, melatonin onset advanced by 28 min in the blue-blocking group compared with the control condition (p=.019). Melatonin levels were significantly higher, favoring the blue-blocking glass condition, at clock time 20:00, 21:00 and 22:00 h, and for sample number 3 and 4. The phase angle (time interval) between melatonin onset and sleep bedtime and sleep onset time increased within the blue blocking group (+45 min and +41 min, respectively), but did not reach statistical significance compared to control condition (+13 min and +26 min, respectively).

**Conclusion:**

Blocking blue light in the evening had a positive effect on the circadian system with an earlier onset and rise of melatonin levels in healthy nulliparous pregnant women. This demonstrated the effectiveness and feasibility of a simple non-pharmacological chronobiological intervention during pregnancy.

## Highlights

• BB-glasses advanced melatonin onset in pregnant women in the third trimester.
• Blocking blue light seems to increase evening melatonin levels in pregnancy.
• BB-glasses may be a treatment to improve circadian function of the pregnant women.

## Abbreviations

ipRGCs: intrincically photo responsive retinal ganglion cells
BB-glasses: blue-blocking glasses
WASO: wake after sleep onset
ANCOVA: analysis of covariance
CI: confidence intervals
DLMO: dim light melatonin onset

## 1 Introduction

Melatonin is a hormone, mainly produced by the pineal gland and has various important biological functions (Tamura et al., 2008). Melatonin acts as a regulator of circadian rhythms, sleep inducer, endocrine modulator, direct free radical scavenger and as a potent anti-inflammatory and antioxidant factor (Lanoix et al., 2008, 2012; Laste et al., 2021; Tamura et al., 2008).

The pineal melatonin production is mainly regulated by the dark-light cycle (Macchi and Bruce, 2004). Melatonin secretions start to rise soon after the onset of darkness, reach maximum levels in the middle of the night and falls before wake-up time (Czeisler and Buxton, 2017). This pattern is normally very stable, making the melatonin profile a reliable phase marker of the circadian rhythm as the main circadian pacemaker, the suprachiasmatic nucleus (SCN), expresses melatonin receptors peaking at the subjective night (Macchi and Bruce, 2004; Masana et al., 2000).

During pregnancy, the night-time melatonin levels increases in the maternal blood after 24 weeks of gestation (Nakamura et al., 2001) and reaches a peak at term (Lanoix et al., 2012). Melatonin crosses all physiological barriers, including blood-brain barrier and placenta (Reiter et al., 2000). Placenta expresses melatonin receptors, indicating that melatonin is involved in the placental function (Lanoix et al., 2008).

Melatonin has beneficial roles in placental and fetal functions and are essential for successful pregnancy (Lanoix et al., 2012; Shimada et al., 2016). In line with melatonin's important biological functions, a diminished maternal plasma and/or saliva melatonin levels are found in complicated pregnancies with preeclampsia (Dou et al., 2019; Lanoix et al., 2012; Shimada et al., 2016) and gestational diabetes mellitus (Laste et al., 2021; Shimada et al., 2016) compared with normal pregnancies. The exchange of melatonin between maternal and fetal circulation is unrestricted, and this circulation provides photoperiodic information to the fetus (Tamura et al., 2008).

Sleep is regulated by circadian rhythms and time spent awake. Hence, disturbances in circadian rhythms often result in disturbed sleep and wakefulness (American Academy of Sleep Medicine, 2014). In this realm, melatonin plays a pivotal role as the hormone transmits information to maintain biorhythms, such as sleep-wake rhythms (Czeisler and Buxton, 2017). Insomnia and other sleep disturbances during pregnancy can affect the perinatal outcomes, and studies indicate that poor sleep increases the risk of preeclampsia, gestational diabetes, perinatal depression, prolonged labor, cesarean birth, intrauterine growth restriction and preterm birth (Ding et al., 2014; Nodine and Matthews, 2013; Palagini et al., 2014).

How well you sleep and how entrained the circadian rhythms are, is closely linked to light exposure. Non-visual effects of light are conveyed by special photoreceptors of the retina, melanopsin-containing intrincically photo responsive retinal ganglion cells (ipRGCs), which via the retinohypothalamic tract, project to the SCN (Buijs and Kalsbeek, 2001). IpRGCs are highly sensitive to a relatively narrow band of wavelengths, specifically to the frequencies between 446 and 484 nm: blue light (Berson, 2007; Brainard et al., 2001). The SCN projects further to the pineal gland, which secrets melatonin (Brainard et al., 2015). Light exposure has an acute suppressive and dose-dependent effect on nocturnal melatonin production (Kayumov et al., 2005; Sasseville et al., 2006; van de Werken et al., 2013). The ipRGC cells also signal both directly and indirectly to brain areas known for its role in regulation arousal and sleep (LeGates et al., 2014). Consequently, artificial light-sources such as smart-phones, computers, tablets and TV with a relative high proportion of blue light illumination, are shown to suppress the melatonin production, increase alertness and delay bedtime (Figueiro et al., 2011; Gronli et al., 2016).

Knowledge of how blue light impact melatonin production in pregnant women has implications for our understanding of the circadian system in pregnancy, as well as clinical relevance. Use of blue-blocking glasses (BB-glasses) in the evening allows the melatonin-production more strongly to follow the natural cycle of light and darkness, even when electric light and light-sources such as smart-phones, computers, tablets and TVs are used (van der Lely et al., 2015). Some studies have already shown that interventions involving blocking the blue light protect endogenous melatonin production from light-suppression at night (Figueiro and Overington, 2016; Sasseville et al., 2006; van der Lely et al., 2015; Zerbini et al., 2020).

Only a few studies have investigated the relationship between light exposure and sleep in pregnant women. One study found that light exposure at night was associated with reduced sleep duration in the first and third trimester (Wada et al., 2012). A more recent study showed that evening light exposure in pregnant women was related to shorter total sleep time and earlier midpoint of sleep as measured by actigraphy (Liset et al., 2021). Early morning bright light therapy for pregnant women with depression, has been found to phase advance the melatonin rhythm (Epperson et al., 2004). A study of healthy night workers in the third trimester of pregnancy showed lower night-time melatonin production in their natural environment, compared to day workers (Nehme et al., 2019). Among night workers an increase in advanced pregnancy and negative offspring outcomes may be related to light-induced suppression of melatonin during night work (Nehme et al., 2019).

Clinical studies on effects of BB-glasses during pregnancy are scarce. One study indicated that BB-glasses may speed recovery from postpartum depression sufferers (Bennett et al., 2009). However, in a previous study, we did not find an effect of BB-glasses on sleep-parameters of healthy nulliparous women (unpublished results). To the best of our knowledge, no study has previously investigated the effect of BB-glasses on melatonin onset, the melatonin profile and the phase angle between melatonin secretion and sleep variables during pregnancy.

Accordingly, the primary objective of the present study was to investigate the effect of blocking blue light in the evening on melatonin onset. The secondary objective was to describe the effect on melatonin profile for clock time and sample number, as well as to investigate the effects on the phase angle (the time interval from a phase marker of the master circadian clock and until another circadian driven event occurs), here assessed by the melatonin onset and bedtime and sleep onset time. We hypothesized that blocking of blue light would result in advancement of melatonin onset. Any effect on the phase angle between melatonin onset and bedtime and sleep onset time was examined.

## 2 Method

### 2.1 Trial design

This study was part of a double blinded randomized placebo-controlled trial, registered at ClinicalTrials.gov (NCT03114072). The trial investigated an intervention to improve sleep, and mood and to increase evening melatonin secretion in pregnant women in the third trimester. In the current study melatonin was sampled from saliva.

The trial was conducted over three consecutive weeks, one baseline week followed by two intervention/control weeks.

### 2.2 Participants

Participants were healthy nulliparous women, recruited between May 2017 and April 2019, during their standard health control (checkup) about 24 gestation weeks. Consulting midwives at antenatal-healthcare centers in the Municipality of Bergen, Norway, mediated the recruitment and provided information about the study (oral and written form) to the relevant participants. If the pregnant women consented to receive more information or participate, further information was provided by the researcher (first author). Inclusion criteria were: 1) nulliparous women, 2) expecting one child, 3) being in the third trimester of a normal pregnancy, 4) able to wear an actigraph during daytime and nighttime for all three weeks (results reported in another paper) and, 5) able to complete questionnaires in Norwegian (results reported in another paper). We recruited nulliparous pregnant women exclusively in order to minimize the risk of their sleep being disturbed by older offspring and to ensure that the participants represented a relatively homogenous group. Exclusion criteria were: 1) somatic or psychiatric disorders, 2) fever and other health conditions affecting sleep, 3) working nights during the study protocol 4) having a condition affecting the translucency of the eyes or 5) melatonin concentrations out of range (either all vaues below 3.0 pg/mL or above 4 pg/mL. The red reflex of both eyes was assessed (McLaughlin and Levin, 2006) to be able to exclude women with serious eye-conditions affecting translucency. The participating pregnant women started the data collection between pregnancy week 27–32, mean week 29+0 days.

Self-reported questions were used to obtain information about maternal age, marital/partner status (married/cohabitating, single, separated/divorced, widow), level of education (high school and below, college and above), income (NOK<600 000, NOK >600 000; 10 NOK ≈ 1 US $), number of people living together in the household (partner, parents, parents in law, children, none, other), smoking (daily, less than daily, never), physical- and relaxing activity.

### 2.3 Interventions

**Fig. 1:**
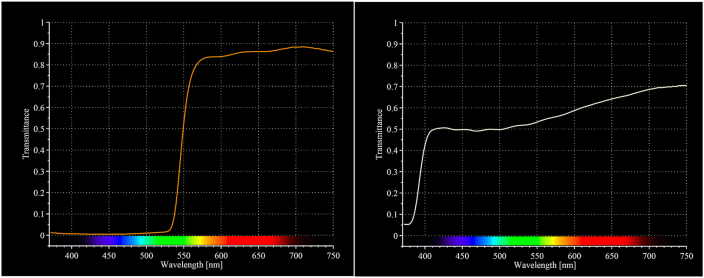
Irradiance spectra from intervention- and control glasses. Note the near complete filtering of blue light spectral irradiance (<530 nm) of the BB-glasses. (For interpretation of the references to color in this figure legend, the reader is referred to the Web version of this article.)

The intervention group wore BB-glasses (Uvex Skyper S1933X, by Honeywell, Smithfield, RI, USA. www.uvex.us) blocking 99% of wavelengths shorter than 530 nm, and approximately 15% of the remaining light spectrum. Participants in the control group wore light grey glasses (Uvex Skyper S1905, by Honeywell, Smithfield, RI, USA. www.uvex.us) blocking approximately 50% of wavelengths shorter than 530 nm, and about 30–50% of light in the remaining visual spectrum. We assessed the light blocking capacity of the BB-glasses and the control glasses by transmittance measurements, using a Ramses hyperspectral radiometer from Trios and a Lions xenon light source. In case of knowledge of BB-glasses a partial blue light blocking condition was used as a control, assumed to maintain the placebo effect. Fig. 1 illustrates the irradiance spectra for the respective glasses. The participants were instructed to wear the glasses from 3 h before normal bedtime at night, and until they turned the lights off to go to sleep. If they were exposed to light at night, such as by going to the bathroom etc., they were instructed to wear the glasses on these occasions also, until final awakening in the morning.

The participants were informed to contact the research team if they experienced any side effects after start wearing the glasses, and they were probed for side-effects after study completion.

### 2.4 Outcomes

The primary outcome was melatonin onset (≥4 pg/mL) measured by saliva samples. The secondary outcome was phase angle (change in latency) between melatonin onset measured by saliva samples and bedtime and sleep onset time assessed by a sleep diary, and melatonin profile for clock time and sample number.

#### 2.4.1 Subjective measure of sleep

A sleep diary was completed every morning. The sleep diary included items on number and duration of naps during the day, use of sleep medication (yes/no), bedtime, lights-out time (trying to sleep), sleep latency, number of nocturnal awakenings, wake after sleep onset (WASO), waking and rise time. Included were also items assessing sleep quality and daytime sleepiness (Carney et al., 2012). The variables used in the present study were bedtime, lights-out time and sleep latency. Sleep onset time was calculated as lights-out time added to sleep latency. The phase angle was calculated as the difference in time between melatonin onset (based on saliva sampling) and bedtime (from sleep diary), and between melatonin onset and sleep onset (from sleep diary), for day 7 and day 21, specifically.

#### 2.4.2 Objective measure of melatonin

Salivette® tubes (Sarstedt AG&Co, Nümbrecht, Germany) were used to collect salivary samples. The participants were instructed to avoid bananas and chocolate the whole sampling day, and in addition to totally avoid drinks with artificial colors, alcohol, caffeine, chewing gum, use of lipstick/lip gloss and tooth brushing during the collection period. They were further recommended not to eat anything during the collection time. In case they needed to eat they were instructed to eat and/or drink right after a sample was taken and then wait for at least 15 min before next sampling.

Saliva was sampled at baseline (day 7) and posttreatment (day 21), at 30-min intervals from 3 h before normal bedtime, overall ranging from 6pm to 2am. Hence, sample number 1 was sampled 3 h before planned bedtime. Participants labeled the samples with id number, date and clock time, and stored the samples in their domestic refrigerator before delivery to a member of the research team, who stored the samples at −70 ֯C.

Melatonin-onset was defined as the time the rising concentration curve crossed 4.0 pg/mL in saliva, using linear interpolation between adjacent samples or linear extrapolation if melatonin levels was between (≥) 3.0 pg/mL and >4.0 pg/mL (Keijzer et al., 2011; Pandi-Perumal et al., 2007). Due to missing data, melatonin onset was estimated for 47 participants. For baseline (N=19) and posttreatment (N=13) participants had melatonin concentrations out of range (either all values below 3.0 pg/mL or above 4 pg/mL).

Melatonin levels were presented by melatonin profile at different clock times and sample number.

Samples were analyzed with enzyme-linked immunosorbent assay (ELISA) kit (EK-DSM, Bühlman Laboratories, Schönenbuch, Switzerland), using a Wallac 1420 Multilabel counter (PerkinElmer Inc., United States) and software Workout 2.5. The analytical sensitivity of this kit was 0.5 pg/mL, and the functional sensitivity was 1.6–20.5 pg/mL, with an inter-assay coefficient of variation of 1.5–6.0 pg/mL for the low and 4.6–18.3 pg/mL for the high controls (PerkinElmer Inc., United States).

Power analysis was calculated prior to the study. The power analysis was based on a 2 x 2 ANOVA analysis where one factor reflected a repeated measure, time (pre vs. post) and where the second factor, condition, was a between group factor (BB-glasses vs. control glasses) and where the relevant outcome comprised the time-by-condition interaction. The estimated sample size needed was based on effect sizes reported in previous studies that have used BB-glasses as intervention and melatonin onset as outcome. For urine melatonin a study showed strong effects on melatonin onset in a group of healthy adults (Ayaki et al., 2016). We expected a medium effect size (Cohens d = 0.50) for the BB-intervention. Setting the alpha to .05 (two-tailed), power to .80, the correlation between repeated assessments to 0.50 revealed that a minimum of 34 participants in total were needed to detect statistically significant time (pre vs. post) x group (BB vs. control condition) interaction effects (Faul et al., 2007).

### 2.5 Randomization and blinding

The participants were randomly assigned by www.randomizer.org to either the intervention (BB-glasses) or control condition (grey glasses). A research assistant packed the glasses into opaque brown paper bags, and by using the randomization key a unique number was created, unknown to the researchers. Condition was revealed to the first author not until the participants were handling in the saliva samples. All participants received the same information about the purpose of the study (two types of glasses filtering different wavelengths of light, and the hypothetical impact on sleep and mood). They were instructed to refrain from researching the topic of light and sleep, and in case they needed to contact the research team not to describe their glasses. Participants with knowledge of BB-glasses were not excluded, since both glasses eliminated some wavelengths shorter than 530 nm. This way we regarded that the placebo-effect was preserved also in the cases of some previous knowledge on effects of blue-filtering devices.

### 2.6 Statistical methods

All statistical analyses were performed using IBM SPSS Statistics version 25 (SPSS, Chicago, IL, USA) and R version 3.5.1 (R Core Team, 2018) and Stata IC version 16 (StataCorp, College Station, TX) for windows.

Characteristics of the study participants are presented as means and standard deviations or numbers and percentages as appropriate.

Melatonin onset was measured at baseline and posttreatment. To examine the effect of BB-glasses on melatonin onset, we used ANCOVA (analysis of covariance) by including the baseline melatonin onset measure as a covariate in linear regression models. The effect estimates were calculated as the difference in melatonin onset means with 95% confidence intervals (CI) between the BB-group and control group, reported in terms of hours and minutes. To further investigate the change in baseline and posttreatment measures of melatonin onset within the BB-group and control group, separately, we used paired *t*-test.

The abovementioned ANCOVA and paired *t*-test analyses were also performed for bedtime and sleep onset, in addition to the secondary outcome, which entailed the phase angle between melatonin onset and bedtime and sleep onset time, respectively, baseline and posttreatment, specifically.

To investigate how salivary melatonin varied by evening hours and sample numbers for both the BB-group and control group, we performed generalized additive models for evening hours and cubic splines regression for sample numbers. The estimated regression lines with corresponding observations points are presented in a graphical format. Because melatonin measures were strongly right-skewed, the regression analyses were performed on log-transformed values to improve model fit.

To test for group difference in salivary melatonin at each evening hour and sample number, we used the Mann-Whitney *U* test. Melatonin values were rounded to nearest hour and observations before 18:30 h and after 24:30 h were excluded (n = 13) due to low numbers.

### 2.7 Ethical considerations

The Regional Committee for Medical and Health Related Ethics, in Western Norway, approved the study (2016/1394/REK vest). All participants included in the study provided written informed consent. After completed participation, all participants were debriefed about the aim of the study, and they were also offered BB-glasses as a compensation.

## 3 Results

**Fig. 2:**
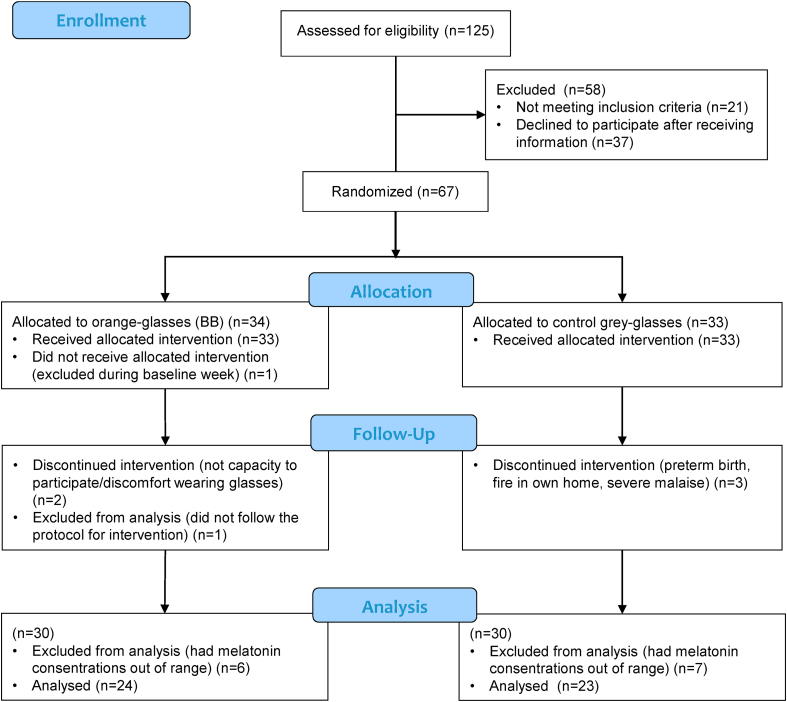
CONSORT 2010 Flow Diagram of enrollment of pregnant women in the study.

Fig. 2 presents a flowchart of study enrollment. In total, 125 pregnant women were assessed for eligibility. After adherence to the inclusion and exclusion criteria and following elimination of those who refused to participate, a sample of 60 pregnant women were analyzed (BB-group n=30, control group n=30). The reasons for exclusion of 7 participants were: preterm birth, fire in own home, severe malaise as a side-effect of the glasses, allergic reaction to the actigraph, not capacity to participate and discomfort wearing glasses. Due to melatonin concentration out of range (either all values below 3.0 pg/mL or above 4 pg/mL) a total of 13 women (6 from BB and 7 from control) were excluded from the final analysis, thereby 24 pregnant women in the BB-group and 23 pregnant women in the control group were included in the final analysis.

Sample characteristics for each group are presented in Table 1. The mean age for the BB-group was 30.0 (SD 3.7) years and 31.0 (SD 4.2) years for the control group. Overall, 96.7% of the nulliparous women were married or living with a partner, 83.3% had education at college level or above, 83.4% had an income of 600 000 NOK (≈60 000 US $) or more. Only one pregnant woman reported she was smoking. None of the pregnant women reported consumption of alcohol during the study weeks.

**Table 1:** Demographic factors for the blue-blocking- and control-group.

Characteristics	Both groups, total	Blue blocking group	Control group
N	60	30	30
**Age, mean (SD)**	30.5 (4.0)	30.0 (3.7)	31.0 (4.2)
**Marital status, N (%)**			
Married/Cohabitating	58 (96.7)	30 (100%)	28 (93.3%)
Single	2 (3.3)	0	2 (6.7%)
**Education, N (%)**			
≤Senior high school	10 (16.7)	6 (20%)	4 (13.3%)
College and above	50 (83.3)	24 (80%)	26 (86.7%)
**Income, N (%)**			
<600 000 NOK	10 (16.7)	5 (16.7%)	5 (16.7%)
>600 000 NOK	50 (83.4)	25 (83.3%)	25 (83.3%)
**Adult, total in household, N (%)**			
1	2 (3.3)	0	2 (6.7%)
2	57 (95.0)	29 (96.7%)	28 (93.3%)
4	1 (1.7)	1 (3.3%)	0
**Children, total in household, N (%)**			
0	58 (96.7)	30 (100%)	28 (93.3%)
1	1 (1.7)	0	1 (3.3%)
3	1 (1.7)	0	1 (3.3%)
**Smoking, N (%)**			
Daily	1 (1.7)	1 (3.3%)	0
Not at all	59 (98.3)	29 (96.7%)	30 (100%)
**Physical activity per day (min), mean (SD)**	23.8 (33.8)	29.7 (39.0)	18.0 (26.5)
**Relaxing activity per day (min), mean (SD)**	5.9 (18.0)	7.0 (19.5)	4.8 (16.4)
**Pregnancy week, mean (SD)**	29.1 (1.2)	28.9 (1.1)	29.3 (1.3)

N=Number of participants; SD = standard deviation; NOK=Norwegian kroner; 10 NOK ≈ 1 United States dollar (US $).

Only two participants reported they had some previous knowledge about the effect of BB glasses.

Table 2 shows the effect of BB-glasses and control glasses on the primary outcome melatonin onset, as well as the secondary outcomes phase angle melatonin onset and bedtime and sleep onset time, in addition to bedtime and sleep onset time. The BB-group showed a significant (p=<.001) advance in melatonin onset by 43 min from baseline to posttreatment period, while the control group with grey glasses showed a significant (p=.002) advance by 11 min. Using ANCOVA with adjustment for baseline values, we detected an estimated 28 min group difference at posttreatment (p=.019).

**Table 2:** Effect of blue-blocking glasses on melatonin onset and phase angle.

Outcome	Blue blocking group		Control group		Estimated group difference (95% CI)^a^	P value
	N	Mean (SD)	N	Mean (SD)		
**Melatonin onset**						
Baseline	23	21:50 (00:56)	18	21:42 (00:49)		
Posttreatment	24	21:07 (01:06)	23	21:31 (00:47)	0 h 28 (0 h 05, 0 h 51)	0.019
P for change^b^		<0.001		0.002		
**Bedtime**						
Baseline	23	23:04 (00:55)	18	22:54 (00:56)		
Posttreatment	24	23:05 (01:03)	24	22:53 (00:51)	−00:02 (−00:36, 00:31)	0.87
P for change^b^		0.81		0.98		
**Phase angle bedtime (in relation to melatonin onset)**						
Baseline	23	1 h 12 (0 h 50)	18	1 h 11 (1 h 02)		
Posttreatment	24	1 h 57 (0 h 55)	23	1 h 24 (1 h 02)	−0 h 22 (−0 h 58, 0 h 14)	0.23
P for change^b^		0.007		0.11		
**Sleep onset**						
Baseline	23	23:54 (01:04)	18	23:33 (01:01)		
Posttreatment	24	23:51 (01:10)	24	23:48 (00:57)	−00:03 (−00:42, 00:36)	0.89
P for change^b^		0.65		0.52		
**Phase angle sleep onset (in relation to melatonin onset)**						
Baseline	23	2 h 03 (0 h 57)	18	1 h 51 (0 h 56)		
Posttreatment	24	2 h 44 (1 h 07)	23	2 h 17 (0 h 58)	−0 h 20 (−1 h 03, 0 h 22)	0.34
P for change^b^		0.037		0.065		

N = Number of participants; SD = standard deviation; CI = confidence interval; phase angle bedtime = time interval from melatonin onset to bedtime (derived from self-reported data); phase angle sleep onset = time interval from melatonin onset to sleep onset (derived from self-reported data). Baseline = day 7, Posttreatment= day 21.

a By ANCOVA.

b By paired sample *t*-test.

**Fig. 3:**
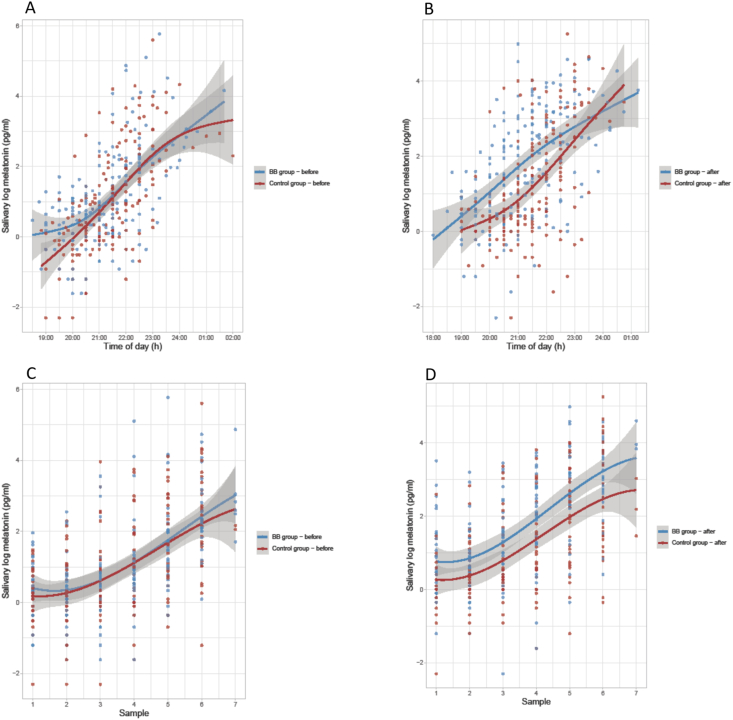
Difference in saliva log-melatonin at clock time and sample number between blue blocking- and control group, at baseline and posttreatment. Log-melatonin visualized for clock-time for (A) baseline and (B) posttreatment, and for sample number for (C) baseline and (D) posttreatment. Median salivary melatonin for each group at each clock time and sample number with a corresponding test for difference are shown in Supplemental Table 1. (For interpretation of the references to color in this figure legend, the reader is referred to the Web version of this article.)

Fig. 3 visualizes the log-salivary melatonin measures for BB group and control group according to clock time and sample number. In Supplemental Table 1 we also present median salivary melatonin for each group at each clock time and sample number with a corresponding test for difference. For clock time at baseline there were no difference between the groups, except at 20:00 h, which amounted to a statistically significant group difference (p=.034). Posttreatment, the evening rise in median melatonin levels were significantly higher in the BB-glasses than in the control condition at 20:00, 21:00 and 22:00 h with p-values of .001, .003 and <.001, respectively.

For sample number, the melatonin profiles showed no statistically significant difference between the groups, at baseline. At posttreatment, sample number 3 (p=.038) and 4 (p=.025) showed higher median melatonin levels for BB-glasses, compared with the control-glasses.

The phase angle between melatonin onset and bedtime and sleep onset time did not differ significantly between the two groups, according to the ANCOVA. However, from baseline to posttreatment, the BB-group showed an increased difference in 45 min for the phase angle between melatonin onset and bedtime (p=.007) and an increased difference of 41 min for the phase angle between melatonin onset and sleep onset (p=.037). The control group showed similar trends, albeit not significant changes from baseline to posttreatment.

The participants reported side-effects for both the intervention and control glasses. The BB-group reported: malaise (n=2) restored after about 5 min, and headache, anxiety and depressive mood (n=1) which lasted for 1.5 h the first evening and 30 min the second evening while still wearing the glasses, and absent the third night. The control group reported severe malaise (n=1) in such a way that exclusion was necessary; headache (n=1) the first evening, then restored; experienced watching double text/subtitles on the TV some evenings (n=1).

## 4 Discussion

The aim of this study was to investigate the effect of blocking the blue light in the evening on melatonin onset, to investigate the association of melatonin profile with clock time and sample number and phase angle between melatonin onset and bedtime and sleep onset time, among nulliparous women in the beginning of the third trimester of the pregnancy.

The results showed that use of BB-glasses advanced melatonin onset. Melatonin profile at clock time and sample number showed a significant group difference for some of the samples, emerged at posttreatment, in favor of the BB-group. Further, the BB-glasses increased the phase angle (time) between melatonin onset and bedtime and sleep onset time within the BB-group, but not in the control group. However, the difference between the groups at posttreatment, adjusted for baseline values, for the two-phase angle variables were not significant.

### 4.1 Melatonin onset

The main objective of this study was to test the effectiveness of blue-blocking glasses on melatonin onset. The melatonin onset for the BB-group advanced by 43 min from pre-to post-intervention compared to 11 min advance in the control group. The group difference at post treatment in melatonin onset, adjusted for baseline values, was significant with 28 min advance in the BB-group.

The current findings are in line with previous research showing acute melatonin suppressive effect of nocturnal light exposure (Kayumov et al., 2005; Sasseville et al., 2006; van de Werken et al., 2013). Dim light melatonin onset (DLMO) is a marker of circadian phase, and by blocking the blue light from reaching the ipRGCs in retina the melatonin-production allows more strongly to follow the natural cycle of light and darkness, even when artificial light and electronic light-sources are used (Sasseville et al., 2006; van der Lely et al., 2015).

As expected in the present study, blocking the blue light in the evening led to an earlier secretion of melatonin in the BB-group, of 28 min earlier melatonin onset compared to the control group (p=.019). The participants in the control group used glasses with partial blue-blocking properties, which resulted in some melatonin protective effect.

The results from this first study on effect of BB-glasses on melatonin outcomes for pregnant women are mainly in line with previous research involving BB-interventions (Ayaki et al., 2016; Figueiro and Overington, 2016; Sasseville et al., 2006; van der Lely et al., 2015; Vethe et al., 2021; Zerbini et al., 2020). In a recent RCT with 12 healthy adults who resided in an evening blue-depleted light environment for 5 days, the melatonin onset (DLMO) was advanced compared with group in usual indoor light (p=.008) (Vethe et al., 2021). In addition, a RCT with 38 young adults, with two weeks intervention, similar to the present study, investigated use of BB-glasses in the evening and showed that BB-glasses significantly advanced melatonin onset (DLMO) compared to the control group with clear lenses (p=<.05). Surprisingly, this only led to advance in melatonin onset (DLMO) in the first but not in the second week of intervention (Zerbini et al., 2020). In contrast to our finding of advanced melatonin onset in the BB-group, in a crossover study with 14 male teenagers, there was no significantly different melatonin onset (DLMO) (p=.351) between the groups (BB-glasses and clear lens-glasses) (van der Lely et al., 2015). The same study however demonstrated a sharper rise of melatonin in the BB-group.

### 4.2 Melatonin profile at clock time and sample time

The melatonin profile for clock time at posttreatment showed a statistically significant difference between the compared groups, suggesting a larger increase in the melatonin level in the BB- group at 20:00, 21:00 and 22:00 h relative to the control group. However, at baseline the difference reached a significant level only at 20:00 h, favoring the BB-group. The pregnant women in the present study were instructed to use BB-glasses from 3 h prior to bedtime, thus the melatonin samples were not measured at the same clock time (range 18:00 to 02:00 h) for all subjects.

In contrast to sample per clock hour, sample number was similar for all the women. Melatonin profile at posttreatment for sample number 3 and 4 showed a statistically significant group difference, indicating a relatively larger increase in the melatonin level in the BB-group. At baseline it did not appear a difference between the two groups.

### 4.3 Phase angle

A second aim of the study was to estimate the effect of blocking the blue light on phase angle with respect to the time lag between melatonin onset and bedtime and sleep onset time. A statistically significant increase within the BB group were shown (p = .007 and p = .037, respectively). A similar pattern was not shown within the control group, and this were not significant, nor the group difference at posttreatment adjusted for baseline values.

The fact that the difference for the two phase angle variables remained non-significant may reflect that the participants could sleep ad libitum, as previous studies have shown that the relationship between melatonin onset and sleep onset is stable, even when a specific bedtime is enforced (Sletten et al., 2010).

Also, these findings are similarly to results published by Van der Lely (van der Lely et al., 2015) where the phase angle between melatonin onset (DLMO) and sleep onset did not significantly differ between the BB- and the control group. However, Zerbini et al. (2020) showed a significantly decreased phase angle between melatonin onset (DLMO) and sleep onset in the intervention group (p=<.05), but only for the first of two intervention weeks.

A recent study showed that in the same sample as the present study evening light exposure was inversely related to total sleep time and positively related to earlier midpoint of sleep, measured by actigraphy, at baseline (Liset et al., 2021).

Maternal melatonin levels have been shown to be important for adequate fetal development (Lanoix et al., 2012) due to the exposure to the maternal rhythms such as maternal melatonin, which crosses the placenta barrier unaltered (Seron-Ferre et al., 1993). Complicated pregnancies are associated with a decrease in melatonin levels, thus indirectly affects the fetus with occurrence of intrauterine growth restriction (Dou et al., 2019; Lanoix et al., 2012; Laste et al., 2021; Nakamura et al., 2001; Shimada et al., 2016).

The present study showed that BB-glasses were able to increase melatonin levels in the evening, which is promoting normal development of the maternal pregnancy (Nakamura et al., 2001). Findings from other studies have identified a synchronous process between the maternal and fetal melatonin levels (Reiter et al., 2000; Tamura et al., 2008), which fortunate the fetal development (McCarthy et al., 2019). The present study may serve as basis for recommendation of BB-glasses to pregnant women as an increase of melatonin secretion did occur at some time points in the evening, and due to the importance of melatonin levels both for the pregnant women and the fetal development.

The observed side effects of the BB-glasses (malaise, headache, lowered mood and anxiety) were transient, and occurred in both groups equally (n=3) except for severe malaise experienced by one participant in the control-group which led to drop-out. The frequency of side-effects is in line with what was reported by Henriksen (Henriksen et al., 2016), while other studies have reported no side effects (Burkhart and Phelps, 2009). These findings are consistent with the previous conclusions from the literature that BB-glasses are a safe intervention when used in the evening and night (Esaki et al., 2017; Henriksen et al., 2016; Shechter et al., 2020). The side effects in five of the six cases disappeared with further use. This observation is not previously reported in similar studies involving BB-glasses and are thus a clinically valuable notion.

Blue light-rich environment at night is not natural and by using BB-glasses one may simulate natural nocturnal light conditions. BB-glasses are inexpensive, safe, and easy to use.

### 4.4 Limitations

The sample in the present study included only 30 participants in each group, which may entail a limitation. After exclusion of 13 participants due to melatonin levels out of range, there were 24 pregnant women in the BB-group and 23 pregnant women in the control group, which may be a further limitation as some uncertainties remain regarding melatonin alternations in the sample as a whole. In addition, the majority of the participants were married or cohabitating, had higher education and income than the general population and they reported good sleep (Liset et al., 2021), which may put limits on the generalizability. On the other hand, the pregnant women could freely follow their rhythms, sleep ad libitum and the saliva melatonin samples were self-collected at home. This may be viewed as an asset in terms of generalizability.

Because we wanted to study the effects of BB-glasses and grey control glasses on melatonin profile as compared to the endogenous melatonin in the naturalistic home-based setting, we could not use the ordinary convention of establishing internal circadian rhythm in the participants; the dim light melatonin onset (DLMO). The DMLO protocol involves use of specialized dark sunglasses prior to and during saliva sampling, which would compromise our study design. The consequence was that we do not have a fixed time point as marker for the internal circadian phase for the participants. Instead, we measured their circadian phase in interaction with the naturalistic light exposure at baseline, and with filtered light exposure during the experimental conditions. In this regard however, it should be noted that some previous comparable studies neither followed the conventional DLMO protocol (van der Lely et al., 2015; Zerbini et al., 2020), or replaced dark sunglasses by BB-glasses (van der Lely et al., 2015). Some participants went to bed before natural rise in melatonin concentration (values > 3.0 pg/mL), which resulted in exclusion from analysis, and this may have affected the results.

It is conceivable that self-report data may lead to recall bias (Coughlin, 1990), social desirability bias (Dodou and de Winter 2014) and some common method bias (Podsakoff et al., 2003). However, the randomization processes would prevent such biases to influence the results. In addition, the melatonin data were derived from saliva samples, and hence not influenced by the previously mentioned biases.

A strength of the present study is the use of objective measure of saliva melatonin, measured repeatedly during one evening both before and after the intervention. As the melatonin saliva sampling was home-based, inadequate sampling procedure may have occurred. However, the subjects received detailed instructions in terms of sampling procedure and home-based melatonin sampling/assessment are shown to correlate well with laboratory assessment (Pullman et al., 2012).

The control glasses in the present study had a partial blue blocking effect of the light, and the results showed that they had some melatonin protective effect. If the control condition was use of clear glasses, the difference between the groups in terms of melatonin onset, melatonin profiles and phase angle would likely have been larger. By using two active interventions we aimed to maintain the placebo-effect in both groups and strengthen the blinding of participants. Two participants reported previous knowledge of effects of blue light and that these glasses work by filtering out specific wavelengths of the visual spectrum. Future studies on the effect of BB-glasses should use a control condition with a minimal reduction of melanopic lux.

## 5 Conclusions

We found that blue-blocking glasses worn 3 h before bedtime protected the melatonin of pregnant women from evening light suppression. Our findings indicate that BB-glasses may serve as an effective non-pharmacological tool for improving circadian function and health for both mother and offspring.

## Funding

The study was supported by a PhD grant for RL, and a grant for graphical design and printing of questionnaire, all funded by The Faculty of Psychology, University of Bergen, Norway. The melatonin samples equipment and analyses were supported by a grant from the Norwegian Competence Center for Sleep Disorders, Haukeland University Hospital, Bergen, Norway. The funders had no role in study design, data collection and analysis, decision to publish, or preparation of the manuscript.

## Trial registration

The trial is registered at ClinicalTrials.gov (NCT03114072).

## CRediT authorship contribution statement

**Randi Liset:** Conceptualization, Methodology, Formal analysis, Investigation, Writing – original draft, Project administration. **Janne Grønli:** Conceptualization, Methodology, Writing- Reviewing and Editing of the final draft. **Roger Ekeberg Henriksen:** Conceptualization, Methodology, Writing- Reviewing and Editing of the final draft. **Tone Elise Gjøtterud Henriksen:** Conceptualization, Methodology, Writing- Reviewing and Editing of the final draft. **Roy Miodini Nilsen:** Methodology, Formal analysis, Writing- Reviewing and Editing of the final draft. **Ståle Pallesen:** Conceptualization, Methodology, Investigation, and Co-authoring original draft.

## Declaration of competing interest

The authors declare that they have no known competing financial interests or personal relationships that could have appeared to influence the work reported in this paper.
