# Supplementary material for: A randomized controlled trial on the effect of blue-blocking glasses compared to partial blue-blockers on melatonin profile among nulliparous women in third trimester of the pregnancy
Source: Neurobiol Sleep Circadian Rhythms. 2021 Dec 29;12:100074. doi: 10.1016/j.nbscr.2021.100074 (PMC8728098; doi:10.1016/j.nbscr.2021.100074)
Supplement: Multimedia component 2 [file mmc2.docx]

**Supplementary material**

**Table 1. Difference in saliva melatonin at clock time between blue blocking- and control group, before and after intervention.**

| **Period** |  | | **Blue blocking group** | | | **Control group** | |  |
| --- | --- | --- | --- | --- | --- | --- | --- | --- |
| **Hours** |  | | N | | Median (IQR) | N | Median (IQR) | P value |
| Pre |  | |  | |  |  |  |  |
| 19:00 |  | | 10 | | 1.5 (0.8, 2.2) | 7 | 0.9 (0.6, 1.2) | 0.096 |
| 20:00 |  | | 52 | | 1.6 (0.9, 2.6) | 46 | 1.1 (0.6, 1.6) | 0.034 |
| 21:00 |  | | 32 | | 2.0 (1.4, 2.6) | 34 | 1.8 (1.0, 3.2) | 0.714 |
| 22:00 |  | | 61 | | 4.8 (1.9, 9.7) | 63 | 3.4 (1.6,11.4) | 0.465 |
| 23:00 |  | | 13 | | 9.1 (3.0, 60.7) | 17 | 8.7 (5.2, 30.5) | 1.0 |
| 24:00 |  | | 10 | | 13.2 (7.6, 20,4) | 6 | 23.5 (11.9, 54,2) | 0.635 |
| Post |  | |  | |  |  |  |  |
| 19:00 |  | | 12 | | 1.6 (1.0, 2.6) | 6 | 1.6 (1.1, 1.8) | 0.639 |
| 20:00 |  | | 50 | | 2.8 (1.2, 6.8) | 47 | 1.3 (1.0, 2.0) | 0.001 |
| 21:00 |  | | 32 | | 4.1 (1.9, 13.8) | 33 | 1.5 (1.2, 3.0) | 0.003 |
| 22:00 |  | | 54 | | 14.6 (4.8, 24.8) | 69 | 4.4 (1.8, 10.6) | <0.001 |
| 23:00 |  | | 17 | | 21.0 (4.8, 30.6) | 19 | 21.0 (6.3, 37.1) | 0.887 |
| 24:00 |  | | 11 | | 31.0 (13.3, 56.2) | 8 | 25.6 (20.0, 52.2) | 0.778 |
|  |  | |  | |  |  |  |  |
| **Sample** | |  | | N | Median (IQR) | N | Median (IQR) | P value |
| Pre | |  | |  |  |  |  |  |
| 1 | |  | | 30 | 1.4 (1.0, 2.7) | 29 | 1.2 (0.8, 2.1) | 0.359 |
| 2 | |  | | 30 | 1.5 (1.0, 2.2) | 30 | 1.2 (0.7, 2.7) | 0.510 |
| 3 | |  | | 30 | 1.8 (1.0, 2.6) | 29 | 1.4 (1.0, 3.4) | 0.927 |
| 4 | |  | | 30 | 2.4 (1.6, 3.5) | 28 | 2.5 (1.0, 7.0) | 0.901 |
| 5 | |  | | 30 | 5.1 (2.7, 8.8) | 29 | 4.0 (2.0, 18.8) | 0.682 |
| 6 | |  | | 26 | 9.5 (7.4, 20.3) | 29 | 10.2 (3.2, 29.3) | 0.800 |
| 7 | |  | | 6 | 15.2 (12.4, 20.1) | 2 | 8.3 (8.0, 8.5) | 0.286 |
| Post | |  | |  |  |  |  |  |
| 1 | |  | | 30 | 1.7 (1.0, 4.3) | 30 | 1.2 (0.8, 1.8) | 0.062 |
| 2 | |  | | 30 | 1.9 (1.2, 4.1) | 30 | 1.4 (1.0, 2.6) | 0.124 |
| 3 | |  | | 30 | 3.9 (1.8, 6.6) | 30 | 1.5 (1.2, 4.1) | 0.038 |
| 4 | |  | | 30 | 8.2 (3.8, 16.3) | 30 | 2.7 (1.7, 7.4) | 0.025 |
| 5 | |  | | 30 | 16.6 (9.9, 33.4) | 30 | 8.5 (2.3, 25.2) | 0.053 |
| 6 | |  | | 28 | 23.8 (14.5, 41.3) | 30 | 12.2 (4.3, 42.9) | 0.253 |
| 7 | |  | | 3 | 52.1 (49.2, 75.6) | 3 | 8.9 (6.6, 14.8) | 0.100 |

N=Number of participants; IQR=Inter Quartile Range.

Estimated by Mann-Whitney U test.
